# Maternal and Child Nutrition and Oral Health in Urban Vietnam

**DOI:** 10.3390/ijerph16142579

**Published:** 2019-07-14

**Authors:** Debbie Huang, Karen Sokal-Gutierrez, Kenny Chung, Wenting Lin, Linh Ngo Khanh, Raymond Chung, Hung Trong Hoang, Susan L. Ivey

**Affiliations:** 1 Health Research for Action, University of California Berkeley School of Public Health, 2140 Shattuck Avenue, 10th Floor, Berkeley, CA 94704, USA; 2 Mailman School of Public Health, Columbia University, 722 W 168th St, New York, NY 10032, USA; 3 Harvard T.H. Chan School of Public Health, 677 Huntington Ave, Boston, MA 02115, USA; 4 Department of Dental Medicine, Long Island Jewish Medical Center, 270-05 76th Ave, Queens, NY 11040, USA; 5 Faculty of Odonto-Stomatology and Department of Dental Public Health, University of Medicine and Pharmacy-Ho Chi Minh City, 217 Hồng Bàng, Phường 11, Quận 5, Hồ Chí Minh 700000, Vietnam

**Keywords:** nutrition transition, oral health, early childhood caries, mouth pain, bottle-feeding

## Abstract

The global nutrition transition has contributed to child obesity and dental caries in developing countries, including Vietnam. Few studies have described the nutrition and oral health of mothers and children. This a descriptive study of the nutrition and oral health characteristics of a convenience sample of 571 children aged 2 to 5 years and their mothers from 5 urban preschools in Central and South Vietnam. The mothers completed a written survey, and the children received dental exams and weight/height measurements. High rates of bottle-feeding and the consumption of sweets were reported. One in 4 children were overweight/obese. Dental caries increased in prevalence and severity by age—at 5 years, 86.7% of children had tooth decay in an average of 8.5 teeth, and 70.9% experienced mouth pain. Most mothers and children suffered from untreated dental disease. Public health programs should focus on nutrition and oral health promotion, as well as dental treatment from pregnancy and birth onward.

## 1. Introduction

Over recent decades, the global nutrition transition has led to the increased childhood consumption of sugary beverages and carbohydrate-dense processed foods and is associated with increased rates of childhood obesity, type 2 diabetes, and tooth decay or caries [1,2]. The adverse effects of the nutrition transition have been particularly dramatic in developing regions, where increasing obesity co-exists with malnutrition, leading to a “double burden” of malnutrition [3]. 

The global nutrition transition has also led to a pandemic of childhood tooth decay or caries, the most prevalent chronic disease worldwide, affecting 60–90% of children [2]. Early childhood caries (ECC), defined as tooth decay in children under age 6, can cause chronic inflammation, mouth pain, difficulty eating and sleeping, malnutrition, a poor quality of life, and a reduced developmental and educational potential [2,4,5,6,7]. Contributors to ECC include nutritional risk factors such as bottle-feeding, and the frequent consumption of sugary beverages and carbohydrate-dense snacks, as well as risk factors for poor oral health such as maternal oral disease, inadequate tooth brushing, and limited access to fluoride and dental care [2,5,6,7]. While dental public health experts assert that ECC is preventable by limiting the nutritional risk factors and supporting positive oral health measures [2,5,6,7,8], these recommendations lack a widespread implementation in developing regions, including in Southeast Asia [2,9,10,11].

Recent studies from Vietnam have demonstrated adverse consequences of the nutrition transition [12,13]. A 2015 study from urban Da Nang found that 70–80% of preschool-age children consumed sweets between meals on a daily basis; and 71% of 3-year-olds and 91% of 5-years-old drank milk with sugar on a daily basis [14]. A 2007 study of preschool children in Ho Chi Minh City found that 20.5% were overweight and 16.3% were obese [15]. The 2009 publication of the 1999 Vietnam’s National Oral Health Survey found that 85% of children aged 6–17 had tooth decay, a significantly higher prevalence and severity than that observed 10 years earlier [11,16]; and studies from 2015–2018 of children aged 2 to 6 in Vietnam found a prevalence of dental caries of around 90% [17,18,19].

Few studies, however, have explored the combination of maternal-child nutrition, and oral health risk factors and outcomes, for preschool-aged children. This paper presents baseline nutrition and oral health data on a convenience sample of children aged 2–5 years and their mothers/caregivers participating in a preventive school-based study designed to understand child nutrition and oral health.

## 2. Materials and Methods 

### 2.1. Study Design and Population

This is a cross-sectional descriptive study of a convenience sample of 571 children aged 2–5 years and their mothers/caregivers in 5 urban and peri-urban preschools/kindergartens in Central Vietnam (Da Nang) and South Vietnam (Ho Chi Minh City). This study was conducted in collaboration with the University of Medicine and Pharmacy, Ho Chi Minh City, and a non-governmental non-profit organization, East Meets West. This paper reports on data collected in 2011. The data were collected on only one child per family, to generate mother-child pairs. The study protocol was approved by the University of California, Berkeley, Committee for the Protection of Human Subjects (#2011-04-3176). The Vietnam partner organizations reviewed the protocol and agreed to rely on the U.S. Institutional Review Board approval, and we developed and signed a Memorandum of Understanding between the US and Vietnamese institutions affirming a reliance on the UC Berkeley Institutional Review Board approval, and detailing each institution’s responsibilities in the study.

### 2.2. Data Collection

Each school director provided information to parents about the study, and obtained the parents’ written informed consent. The consent form and survey were translated from English to Vietnamese, and back-translated to English to ensure accuracy. The materials were also reviewed and edited by our Vietnamese partners to ensure cultural relevance and an appropriate literacy level. After parents provided written consent, the children were asked to assent, and the following data were collected: 

*(1) Parent survey:* Based on the school directors’ request, written surveys were sent home for parents to complete and return. The parent survey consisted of 50 questions, including family demographics, maternal oral health knowledge, maternal and child nutrition and oral health practices, the child’s complaint of mouth pain, and the parent’s assessment of the child’s oral health and overall health. The survey was adapted from the World Health Organization (WHO) global oral health surveys, with adaptations as requested by the local partners [20].

*(2) Child dental screening exam:* Child dental exams were performed by licensed Vietnamese dentists assisted by a dental nurse or student. The examiners were standardized by local university dental trainers according to WHO standards [20]. The exams were conducted on a designated day in school, in a classroom where several dentists at a time examined children in reclining dental chairs, with a natural light and headlamp, a mirror and a dental probe. The presence of decayed (cavitated), missing (extracted due to decay) and filled teeth was recorded for each child. The decayed, missing, filled teeth (dmft) index is the sum of the number of teeth that are decayed, missing due to decay, and filled due to decay. For children in Da Nang, the depth of cavitation by visual inspection was recorded as into the enamel, dentin, or pulp. 

*(3) Child anthropometric measurements:* Children’s height and weight were measured, in lightweight clothing and without shoes, by trained volunteers using a professional-grade stadiometer and scale (Seca, Chino, CA). The height was recorded to the nearest centimeter and the weight to 0.1 kilogrammes.

### 2.3. Data Analysis

The data were entered into Excel and systematically checked prior to importing into SPSS v.21 (IBM Corp, Armonk, NY, USA) and running descriptive analyses. WHO Anthro (World Health Organization, Geneva, Switzerland) (v.3.2.2, for children up to 5 years of age) and WHO Anthro Plus software (v. 1.0.4 World Health Organization, Geneva, Switzerland) was used to calculate the children’s growth status according to global reference standards based on their age, height, and weight: the height-for-age Z score (HAZ), weight-for-age Z score (WAZ), weight-for-height Z score (WHZ), and BMI-for-age Z score (BAZ) [21]. Children with HAZ, WAZ, WHZ and/or BAZ < −2 were considered “malnourished” (under-nourished). For children under age 5, those with BAZ > +2 and < +3 were considered overweight, and BAZ > +3 were considered obese; for children age 5 and over, those with BAZ > +1 and < +2 were considered overweight, and BAZ > +2 were considered obese [21].

## 3. Results

### 3.1. Demographics

This study involved 571 children aged 2 through 5 years old, and 571 mothers/caregivers. The children’s mean age was 4 years, and approximately half were male and half were female. The mothers had a mean age of 33 years, and nearly 13 years of education. The mean time for participants to walk from home to a store that sold snack food and sugary beverages was 5 minutes (Table 1).

### 3.2. Nutrition

Mothers drank milk a mean of 3 times a week, and drank soda and ate salty snacks slightly less than once a week. Nearly all children (95.3%) were breastfed, for a mean duration of 14.7 months. In addition, 8 in 10 children (83.1%) were bottle-fed, for a mean duration of 19.6 months. Children drank milk a mean of twice a day, approximately five times as often as their mothers. Children also drank soda a mean of 1–2 times per week, and ate sweet snacks and salty snacks twice a week, twice the frequency of their mothers. In total, fewer than 5% of the children experienced malnutrition, and nearly 1 in 4 children (23.2%) were overweight or obese (Table 2).

### 3.3. Oral Health

Although nearly all mothers (98.0%) reported having had at least one dental visit, nearly all mothers (91.1%) reported that they currently suffered from oral health problems, including decayed teeth, inflammation, dental pain, and bleeding gums.

Most mothers knew that childhood tooth decay could be caused by sweets (67.5%) and not brushing teeth (52.7%), however few knew that sugary beverages (5.2%) and the extensive use of the baby bottle (2.7%) could cause decay. Eight out of 10 mothers reported that they helped their children brush their teeth frequently or almost always, and nearly half (44.4%) had taken their child to the dentist.

**Figure 1:**
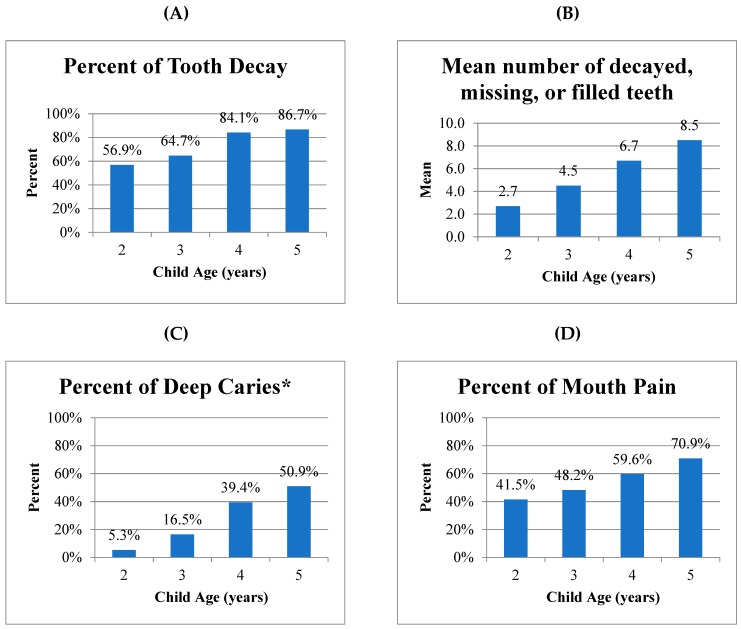
Prevalence and severity of child tooth decay. Note: Sample size: Panel (**A**) (*n* = 571); Panel (**B**) (*n* = 571), Panel (**C**) (*n* = 339 * data from Da Nang only), Panel (**D**) (*n* = 423).

Overall, child dental exams indicated that 3 out of 4 children (74.6%) had tooth decay. Nearly all (96.4%) of the decay was untreated. The prevalence of child tooth decay increased steadily with age from 56.9% at 2 years to 86.7% at 5 years. Likewise, the mean number of decayed, missing, and filled teeth increased steadily with age from 2.7 at 2 years to 8.5 at 5 years (Figure 1). As measured in one of the two regions, 1 in 3 children (29.8%) had deep decay into or near the pulp, by visual inspection (Table 2). The prevalence of deep decay increased steadily with age to affect half of the children (50.9%) at 5 years of age (Figure 1).

Overall, more than half of children (56.3%) complained of mouth pain, 4 in 10 children (40.7%) had problems eating due to mouth pain, and nearly 1 in 4 children (21.9%) had problems sleeping due to mouth pain. The prevalence of mouth pain increased with age, corresponding to the increase in the prevalence and severity of dental caries, and 7 out of 10 children in the study had mouth pain at age 5 (Figure 1). Mothers’ assessment of their children’s oral health was worse than their general health (Table 2).

## 4. Discussion

This sample of families from urban/peri-urban southern and central Vietnam displayed many of the advantages of economic development: high school education, small families, access to food markets, and public drinking water that included fluoridation in some districts of Ho Chi Minh City [22]. Mothers had preserved some healthy traditions, such as breastfeeding their babies for one year, and only rarely consuming soda and junk food themselves. They also reported helping their children brush their teeth, following preventive care recommendations such as prenatal care and child immunizations, and taking their children to the dentist. Child undernutrition—which was, historically, a widespread public health problem in Vietnam—was rare in our study population. 

However, the adverse effects of the global nutrition transition were evident. Families had a quick access to stores that sold heavily-advertised and inexpensive sugary drinks and non-nutritious snack foods. In addition to breastfeeding, most children were bottle-fed to a mean of nearly two years of age, likely related to mothers working outside of the home [23], and to the widespread advertising, affordability, convenience, and modern status of infant formula [24,25]. While children were given milk 2–3 times per day—which has good nutrients—many milk products in Vietnam have added sugar, and many families add sugar to milk in the baby bottle and cup [16,26]. Children consumed soda, sweet snacks and salty snacks twice as frequently as their mothers. This study supports other research that has demonstrated an epidemic of childhood obesity in urban Vietnam [27,28]. 

Regarding oral health, nearly all mothers were suffering from untreated dental and/or gum disease. This could interfere with the mothers’ nutrition and overall health, as well as their caregiving and work capacity. In addition, some studies have associated poor maternal oral health during pregnancy with preterm delivery and low birthweight [29], along with the transmission of maternal oral cariogenic bacteria to their children during infancy, contributing to their children’s severe ECC [30]. Additionally, most children were bottle-fed, for a mean of nearly 2 years, and few mothers knew that sugary beverages, the use of the baby bottle for sleeping, and use past one year of age could contribute to ECC/tooth decay.

Tooth decay was evident at two years of age, and increased steadily in prevalence and severity through age 5, at which time 86.7% of children had tooth decay and 70.9% experienced dental pain. Although half of the families reported taking their child to the dentist, most children had numerous untreated decayed teeth, with 96% of caries untreated. This may indicate parents’ and teachers’ lack of awareness of the importance of the prevention and treatment of caries in children’s primary teeth, and the common belief that baby teeth do not matter because they just fall out [31], which reflects a lack of understanding that early childhood oral health is critical for young children’s nutrition, growth, development, and wellbeing. Even among children who had received dental treatment, it was inadequate to meet their extensive treatment needs. Our findings confirm those of numerous studies that have found that Vietnam and other Southeast Asian countries have among the highest rates of ECC globally [11]. Other recent studies in Vietnam have found a greater likelihood of dental caries associated with a lower parent income, education and knowledge about oral health, a low child birthweight, the male gender, prolonged bottle-feeding and breastfeeding, the frequent consumption of sweets, poor toothbrushing habits, and limited access to dental care [17,18,19]. Many studies have found associations between caries and obesity, likely due to the common dietary risk factors, and the parenting practice of giving children frequent sweet snacks and drinks for behavior control [32]. 

The large increase in the prevalence of ECC from birth to age 2, and the large increase in the severity of ECC and mouth pain from ages 5 to 6 indicate ages at which prevention and treatment interventions are especially needed, for the prevention of both caries and obesity, and to ensure that children are ready to learn in school. Maternal-child nutrition education should promote a family consumption of healthy snacks and beverages, limiting the consumption of junk food and sugary drinks, avoiding milk with added sugar, stopping bottle-feeding after the first birthday, and encouraging healthy parenting and discipline practices that avoid giving infants and toddlers sweet drinks and snacks in response to fussy behavior. In addition, maternal-child dental interventions should include ensuring a maternal dental treatment during pregnancy and a child dental prevention and treatment from infancy onward, as well as teaching parents to help brush their children’s teeth from infancy to 8 years of age, as recommended by pediatric dental authorities [7,8,33].

Attention to caries prevention from pregnancy and birth onward—including nutrition and oral health education, fluoridated water, daily toothbrushing with fluoride toothpaste, fluoride varnish, and silver diamine fluoride—has been shown to substantially reduce ECC and the need for dental surgical treatment [2,7]. Furthermore, environmental supports—such as taxes on sugary drinks and snacks, and prohibiting the sale and consumption of junk food in schools and health settings—can help families make healthier choices, as seen in Mexico and other local areas [34]. Future research and advocacy are needed in low-resource countries to demonstrate that investing in population-wide nutrition, as well as oral health promotion and caries prevention from pregnancy and birth onward can reduce medical and dental costs, and improve children’s nutrition, oral health, educational potential, and future economic capacity.

This study has some limitations. It is a convenience sample of urban/peri-urban children from two geographic areas in Vietnam and may not represent children from other regions, including rural areas where maternal education, economic resources, and access to food and dental care may be more limited. This school-based study served mostly middle-class families with sufficient education and literacy to complete a written survey. As such, the results may not be generalizable to families with less education and fewer resources. As with all cross-sectional studies, causation cannot be determined. The surveys were brief to reduce parent burden, and did not include a full maternal-child diet history. The parents may have underestimated the frequency of their children’s consumption of junk food and sweetened beverages because they were unaware of what their children consumed—particularly older children who may spend less time under maternal supervision, may be given junk food by family or friends outside of the home, and may purchase items on their own. Finally, mothers’ dental symptoms were assessed only by a self-report, without confirmation of their oral health status by examination.

The study has many strengths, in particular forming local partnerships that facilitated relationships with the school sites, the trust of the study population, and efficient data collection. Most child oral health studies focus on school-age children—once caries is a universal and severe problem—and do not address the “upstream” maternal risk factors, such as poor maternal oral health and nutrition, nor infant/toddler risk factors such as bottle-feeding and the consumption of sugary beverages and snacks. This study adds to the literature by identifying maternal-child nutrition and oral health factors in younger children that may be amenable to early preventive interventions.

## 5. Conclusions

This sample of children aged 2 to 5 years old from two urban/peri-urban regions of Vietnam found evidence of the global nutrition transition and its adverse impact on child nutrition and oral health. Most children were bottle-fed in addition to being breast-fed, and were frequently given non-nutritious and sugary snacks and beverages. The population had a high prevalence of child obesity and widespread untreated maternal and child dental disease. The prevalence and severity of dental caries increased steadily from ages 2 to 5, at which time the majority of the children experienced severe tooth decay and dental pain. Public health programs should implement prevention and treatment strategies to improve maternal-child nutrition, oral health and overall wellbeing, from pregnancy and birth onward.

## Figures and Tables

**Table 1:** Demographic characteristics of families, mothers and children.

Characteristics	Number of Respondents (*n* = 571 Families)	% or Mean (SD)
Locations:		
Central Vietnam	339	59.4%
South Vietnam	232	40.6%
Maternal Characteristics:		
Mother Age (years)	553	33.1 (5.0)
Mother Education Level (years)	485	12.8 (3.7)
Child Characteristics:		
Child Age (years)		
2	65	11.4%
3	201	35.2%
4	207	36.2%
5	98	17.2%
Child Sex (% Male–% Female)	287–274	51.2%–48.8%
Household Characteristics:		
Number of children in household	567	1.6 (0.6)
Number of people in household	532	4.5 (1.7)
Home has electricity	563	99.5%
Home has potable water	531	98.7%
Time to walk from home to store selling processed snack food (minutes)	560	5.0 (7.5)

**Table 2:** Maternal and child nutrition and oral health characteristics.

Nutrition Characteristics	Total Number of Respondents	Percent Daily	Mean (SD) or %
**Mother’s Nutrition Practices**			
Frequency of milk consumption	557	27.7 % daily	3.3 (4.4) times/week
Frequency of soda consumption	547	2.7% daily	0.9 (1.6) times/week
Frequency of chips consumption	548	1.3% daily	0.8 (1.3) times/week
**Child Nutrition Practices**			
Breastfed	548		95.3%
Duration of breastfeeding (months)	482		14.7 (7.8) months
Bottle-fed	537		83.1%
Duration of bottle-feeding (months)	155		19.6 (11.7) months
Fell asleep with the baby bottle in mouth			
Occasionally	432		17.6%
Frequently/almost always			10.2%
Frequency of milk consumption	486	91.2 % daily	13.9 (5.8) times/week
Frequency of soda consumption	441	5.7% daily	1.3 (2.8) times/week
Frequency of chips consumption	478	11.1% daily	1.9 (2.7) times/week
Frequency of sweets consumption	462	11.9% daily	2.0 (3.0) times/week
**Child Nutrition Status**			
Height-for-age malnutrition	554		4.2%
Weight-for-age malnutrition	554		2.4%
Weight-for-height malnutrition	477		1.1%
BMI-for-age malnutrition	553		1.5%
BMI-for-age overweight/obesity	553		23.2%
**Oral Health Characteristics**			**Mean or %**
**Mother’s Oral Health Status**			
Mother report of current oral health problem (e.g., pain, decayed tooth, abscess, inflammation, bleeding gums)	425		91.1%
Mother ever visited a dentist	557		98.0%
Time since mother’s last dental visit (months)	377		8.5 (14.8) months
Mother received prenatal care	554		100%
Mother’s Knowledge about Child Oral Health			
Knows that sweets and candy can cause tooth decay	480		67.5%
Knows that sweet drinks can cause tooth decay	480		5.2%
Knows that bottle-feeding can cause tooth decay	480		2.7%
Knows that not brushing teeth can cause tooth decay	480		52.7%
**Maternal-Child Oral Health Practices**			
Child has own toothbrush at home	545		99.1%
Family has toothpaste at home	545		96.0%
Mother helps child brush teeth frequently/almost always	545		80.7%
Child ever visited a dentist	455		44.4%
**Child Oral Health Status**			
Percent of children with any tooth decay	571		74.6%
Average proportion of teeth with untreated decay* (d/dmft)	429		96.4%
Number of teeth with active decay (d)	571		5.5 (5.2)
Decayed, missing, filled teeth (dmft) index	571		5.8 (5.4)
Presence of deep decay near pulp (data from Da Nang only)	339		29.8%
Child complains of pain in mouth/teeth	423		56.3%
Child has problems eating due to mouth pain	533		40.7%
Child has problems sleeping due to mouth pain	538		21.9%
Mother’s assessment of child’s oral health as poor	547		14.8%
Mother’s assessment of child’s overall health as poor	542		2.0%

* “Average proportion of teeth with untreated decay” was calculated by d/dmft: “d” (the number of decayed teeth) divided by the “dmft” (sum of the number of teeth that are decayed, missing [due to decay] and filled [due to decay].
